# Minimally Invasive Surgery for Simple Congenital Heart Defects: Preserving Aesthetics without Jeopardizing Patient Safety

**DOI:** 10.3390/jcdd10110452

**Published:** 2023-11-06

**Authors:** Mauro Lo Rito, Ylenia Claudia Maria Brindicci, Mario Moscatiello, Alessandro Varrica, Matteo Reali, Antonio Saracino, Massimo Chessa, Tommaso Aloisio, Giuseppe Isgrò, Alessandro Giamberti

**Affiliations:** 1 Department of Congenital Cardiac Surgery, IRCCS Policlinico San Donato, San Donato Milanese, 20097 Milan, Italy; ylenia.brindicci@gmail.com (Y.C.M.B.); alessandro.varrica@grupposandonato.it (A.V.); matteo.reali@grupposandonato.it (M.R.); alessandro.giamberti@grupposandonato.it (A.G.); 2 Department of Pediatric and Adult Congenital Cardiology, IRCCS Policlinico San Donato, San Donato Milanese, 20097 Milan, Italy; antonio.saracino@grupposandonato.it (A.S.); massimo.chessa@grupposandonato.it (M.C.); 3 Department of Cardiothoracic and Vascular Anesthesia and Intensive Care Unit (ICU), IRCCS Policlinico San Donato, San Donato Milanese, 20097 Milan, Italy; tommaso.aloisio@grupposandonato.it (T.A.); giuseppe.isgro@grupposandonato.it (G.I.)

**Keywords:** atrial septal defect, partial anomalous venous return, partial atrioventricular septal defect, minimally invasive surgery, right anterior thoracotomy, congenital heart defect, surgery

## Abstract

Minimally invasive surgeries for pediatric patients have been proposed for decades, with different approaches in mind. Minimal right axillary thoracotomy (MRAT), proposed two decades ago, allows the preservation of patients’ safety alongside faster aesthetic and functional recovery. The MRAT did not become widely adopted due to the prejudice that to follow a minimally invasive approach, safety and efficacy must be compromised. With this study, we aim to compare MRAT to the standard median sternotomy approach with a focus on safety and clinical outcomes. Between January 2017 and April 2021, 216 patients diagnosed with ASD, pAVSD, or PAPVD underwent surgical repair with different approaches in the same period. MRAT was used for 78 patients, and median sternotomy was used for 138 patients. In this last group, standard median sternotomy (SMS) was used for 116 patients, while a minimal skin incision (SMS mini) was used for 22 patients. There were no major complications overall nor in each specific approach. MRAT enabled the successful repair of simple heart defects, providing similar post-operative and cardiological recovery. MRAT does not compromise patients’ safety and does not prolong the duration of surgery once the learning curve is overcome, which is generally after 15–20 consecutive operations.

## 1. Introduction

Surgical repair of simple congenital heart defects, such as atrial septal defects (ASD), partial atrioventricular septal defect (pAVSD), and partial anomalous pulmonary venous return (PAPVD), using standard median sternotomy (SMS) has excellent results, with low mortality and morbidity [1,2]. We aim to specify that the treated PAPVD was, above all, with a return to SVC and occasionally to IVC, and the surgical approach was sometimes in minimal right axillary thoracotomy. Still, more often, we treated PAPVD in sternotomy or mini-sternotomy. Considering that we were establishing a program, we decided to use MRAT primarily for ASD for the first few years.

In the last decade [3,4,5], different minimally invasive approaches have been proposed to enhance patients’ recovery and provide better cosmetic results [3,4,5,6,7]. Surgical approaches, such as right anterolateral thoracotomy, posterolateral thoracotomy, and partial sternotomy, may carry the risk of suboptimal results [8,9,10,11] in a growing number of patients. Possible complications related to the approach can be scoliosis and asymmetrical breast development, which are fundamental to patients’ appearance and satisfaction [11,12]. To overcome these drawbacks, the technique of minimal right axillary thoracotomy (MRAT) was developed and introduced as a variation of Denis Browne’s original right thoracotomy [13], allowing correction of simple congenital heart defects while avoiding chest wall deformities and anomalous breast development and hiding surgical scar beneath the right arm. With this study, we aim to assess the results of the introduction of MRAT for simple defects compared to standard approaches (SMS and SMS mini) in terms of surgical outcomes and early results.

## 2. Materials and Methods

In this retrospective single-center study, we included all patients with the diagnosis of atrial septal defect (ASD), partial atrial septal defect (pAVSD), and partial anomalous pulmonary venous return (PAPVD) operated using MRAT or SMS between January 2017 and April 2021 at the IRCCS Policlinico San Donato. All subjects with other diagnoses or those who were treated with other approaches were excluded. Clinical and operative data were obtained from medical records, operative notes, and follow-up reports. Outcomes of interest were operative parameters, post-operative course, incidence of complications, and wound-related complications. We included 216 patients divided according to the approach in the following groups: MRAT (n = 78 pts) and sternotomy (n = 138 pts). In this last group, SMS was used in 116, and in the other 22, we adopted a minimal cosmetic skin incision (SMS mini).

### Statistical Analysis

We expressed continuous variables as median with interquartile range (IQR), considering a non-normal distribution that was assessed with the Shapiro–Wilk test. The differences between groups were assessed with Kruskal–Wallis Test and Dunn’s test with Bonferroni correction to evaluate differences among the three groups. Categorical and ordinal variables were reported as frequencies and percentages, and differences were assessed with Chi-squared or Fisher exact test according to group size. We investigated the improvement or worsening of echocardiographic parameters (intended as a shift from the starting value) between pre-operative and discharge using symmetry test (Bowker’s test and the Stuart–Maxwell test). The Bowker’s test is a symmetry test, and the Stuart–Maxwell test is marginal homogeneity test; if both tests provide significant *p*-value, then a significant shift has occurred. *p*-value was considered significant when <0.05. Data analyses were performed with Stata Statistical Software (Release 17; StataCorp 2022, College Station, TX, USA: StataCorp LP).

## 3. Results

Among the 216 patients, there were 128 females (59.3%) and 88 males (40.7%) with a median age at surgery of 6.1 years (IQR: 4–14.5). The most frequent diagnoses were ASD (n = 149 pts), PAPVD (n = 54), and pAVSD (n = 13). At the pre-operative echocardiogram, the left ventricular function was preserved; meanwhile, the right ventricle had moderate or severe dilatation in 88.8% (see Table 1). Mitral and tricuspid valves presented severe regurgitation in a minority of the population, as described in Table 1.

### 3.1. Surgical Technique Description

**Figure 1:**
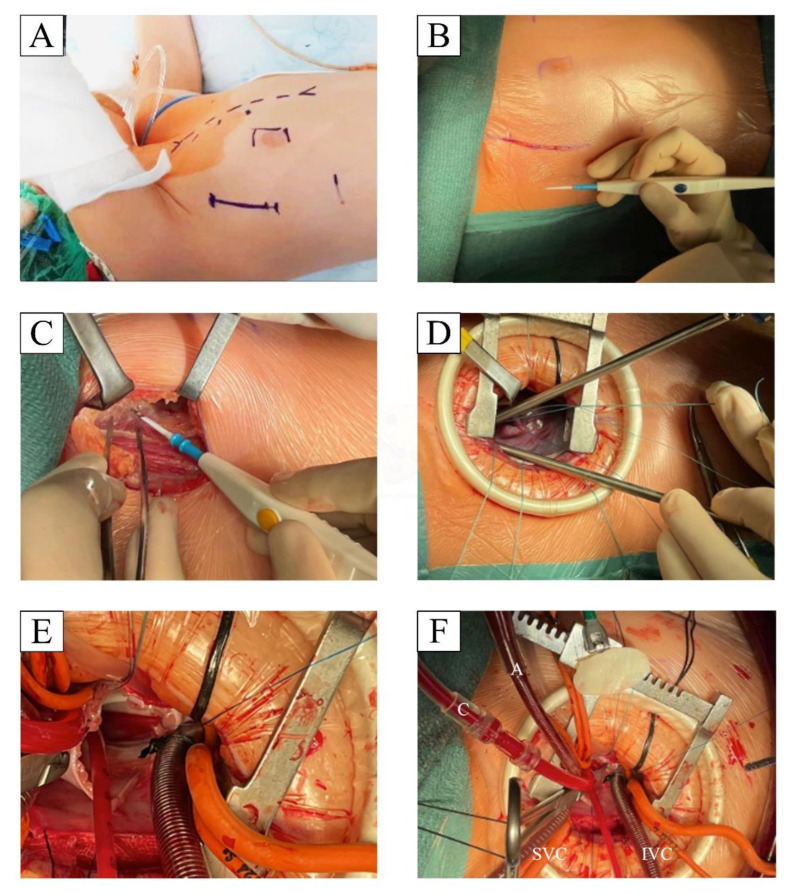
Patient preparation and surgically relevant steps: (**A**) Patient reference marking for MRAT incision, right nipple, and potential bail-out sternotomy; the patient is placed in partial left decubitus with the right arm lifted gently above the head. (**B**,**C**) limited skin incision and muscle sparing with preservation of the long thoracic nerve. (**D**) Soft tissue and finocchietto exposure with pericardial suspension. (**E**) atrial septal defect visualization with central cannulation. (**F**) atrial septal defect patch closure.

All operations, independent from the surgical approach, were monitored similarly with arterial access (preferably right radial artery), right jugular internal central venous catheter, transesophageal echocardiography, and defibrillating external pads for MRAT and SMS mini. The use of external defibrillating pads is indicated for the minimal approaches because it is possible that internal defibrillating palettes may not be used. The cardiopulmonary bypass strategy was similar in all approaches with aortic cross-clamp, cardioplegic arrest, and operation conducted in normothermia or mild hypothermia. We administered antegrade hematic or crystalloid cardioplegia that provided long myocardial protection, avoiding the need for repetition. The SMS approach was prepared as routinely performed. In 2018, we started the program for CHD minimally invasive treatment, and, with regard to MRAT, we adopted the approach described by Prêtre et al. [14] for the patients with partial left lateral decubitus and a 3–5 cm incision in the right anterior axillary line that needed to be hidden beneath the right arm (Figure 1A–C). We usually entered the chest cavity in the fourth intercostal space for ASD and small-size patients (<20 kg) and the third intercostal space for PAVD and larger patients that required direct aortic cannulation (>20 and <50). We modified the Prêtre approach by adding the Alexis wound protector/retractor (Applied Medical) that fit well with different pediatric chest conformations (Figure 1D). We used size S for pediatric cases and size M for adolescent/adult patients. The soft tissue retractor was placed after the opening of the intercostal space and allowed gentle retraction of the wound’s surrounding tissues; the finocchietto spreader was then placed to enlarge the intercostal space (Figure 1D). The soft tissue retractor could not be used by adding a second finocchietto spreader in orthogonal direction to retract the soft tissues. The remaining preparation technique does not differ from the already published method [14]. The cannulation strategy needs to be planned ahead of the case, with proper device selection. In particular, we prefer central cannulation (aortic/bicaval) for all cases weighing <30 kg, a hybrid strategy for cases weighing between 30 and 50 kg, and complete peripheral cannulation for cases >50 kg. In particular, for patients weighing between 30 and 50 kg, the cannulation strategies were personalized to patients, height, and size, considering femoral artery and vein cannulation in patients that already had a developed “adult” body conformation. For adult-sized patients, peripheral cannulation consisted of a typical setting for minimally invasive surgeries, with percutaneous neck right SVC cannulation and surgically isolated femoral vessel direct cannulation. After decannulation, the femoral artery, especially for adolescents or borderline weight cases, was reconstructed with a pericardial patch to avoid stenosis related to purse string closure.

### 3.2. Demographic Data and Operative Results

The sternotomy (SMS) was the approach most frequently used (63.9%) with a subgroup of patients in which we made a 5 cm skin incision (22/138) but performed a full sternotomy (SMS mini). The most frequent operation performed with SMS was ASD patch closure (56%), followed by PAPVD repair (36%) and pAVSD correction (8%). We used SMS mini only for ASD closure (n = 17) and PAPVD repair (n = 5), and we did not treat any case of pAVSD. Main echocardiographic preoperative findings showed typical characteristics for an interatrial shunt with a volume overload of the right ventricle with moderate-to-severe dilatation in 89% of the cases and moderate-to-severe tricuspid valve regurgitation in 11% of the population. Detailed information on the population according to the approach is illustrated in Table 1.

We began a minimally invasive structured program in 2018 with the adoption of the MRAT approach. The minimally invasive program was planned with the aim of abolising the surgical learning curve and eliminating any potential complications. We invited an experienced surgeon (R. Prêtre) to perform the first cases, teaching everyone relevant methods and warning us of potential pitfalls. The first year of the program focused on only performing ASD cases in the weight range of (<20 kg), which allowed central cannulation to maintain a homogeneous and replicable method among the four surgeons of the team who received equal and regular case exposure. After the first year, we widened our indications including differents diagnosis and patients’ weight, and the MRAT rapidly outgrew the SMS cases (34 vs. 24) in the second year of the program. The cannulation strategies adopted are detailed in Table 2. In the MRAT group, the most frequent defect repaired was ASD (85.9%—n = 67), with few cases of PAPVD (8.9%—n = 7) and pAVSD (5.1%—n = 4). Due to this initial program plan, the adoption of MRAT was significantly more frequent for ASD (85.9% vs. 77% vs. 56% *p* < 0.001) compared to the SMS that was preferred in PAVPD and pAVSD.

**Figure 2:**
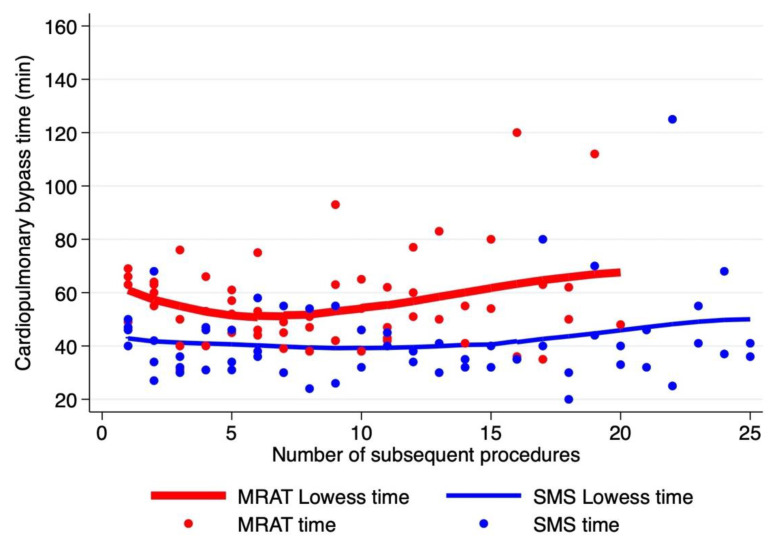
Time of cardiopulmonary bypass time according to the number of procedures starting from the first operation. MRAT (in red dot and line) vs. SMS (in blue dot and line). Lines represent Lowess cardiopulmonary bypass time for each approach (locally weighted scatterplot smoothing).

**Figure 3:**
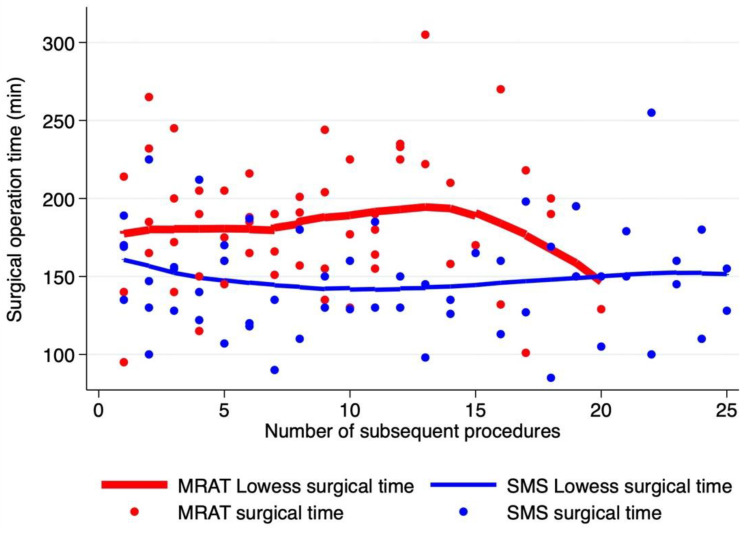
Time of surgical operation, determined as skin-to-skin time (min), according to the number of procedures starting from the first operation. MRAT (in red dot and line) vs. SMS (in blue dot and line). Lines represent Lowess surgical time for each approach.

In terms of operative details, the patients of the MRAT group were significantly younger and were lighter in weight and smaller in height compared to the SMS (Table 3). There was no significant difference in relation to cardiopulmonary bypass (Figure 2) and aortic cross-clamp, although surgical times were significantly longer for the MRAT compared to SMS (190 vs. 170 min, *p* = 0.0013). Accounting for the learning curve, the analysis of surgical times among the four surgeons showed that for MRAT, the surgical operative times become comparable to SMS after 15–20 procedures (Figure 3), indicating that longer surgeries were due to initial experience with this new approach. The majority of MRAT patients had only one chest drain (75/78), draining both the pleural and pericardial spaces. Meanwhile, in the SMS patients, a minority had only one drain (27/116) and SMS mini (15/22). The median ICU stay was 1 day for every approach without any significant differences. The overall median hospital stay was 7 days (IQR: 6–9) with no differences among the three approaches [MRAT 7 days (IQR: 6–7)–SMS 7 days (IQR: 6–10) days, SMS mini: 6 days (IQR: 6–7 days)]. The pericardial effusion of any entity occurred more frequently in the SMS compared to the MRAT (18.8% vs. 2.6%, *p* = 0.001), although all were treated medically except for four cases in the SMS that required subxiphoid pericardial drainage before discharge. In 1 MRAT case, we observed, at pre-discharge echo, the presence of a para right atrial clot without hemodynamic impact that gradually resolved in the follow-up.

### 3.3. Echo at Discharge

A minimal, insignificant residual defect was observed in 10 cases (4.6%). After PAVPD double patch repair, five patients (SMS = 3, MRAT = 2) had isolated non-significant gradients (<5 mmHg) between the superior vena cava and right atrium and did not require any intervention during the follow-up. Trivial residual interatrial shunts on the ecodoppler were seen, pre-discharge, in four MRAT cases and were not appreciable at the first echocardiography follow-up. In one SMS mini case, a significant SVC to right atrium gradient was observed after the Warden operation for PAPVD that required balloon dilatation and did not require any further intervention.

The post-operative echocardiography showed a typical trend of repaired ASD and PAVPD with a reduced degree of RV dilatation (*p*-value > 0.001), improvement in the degree of TV regurgitation (*p*-value > 0.001), and reduced pulmonary artery pressure measurements (*p*-value > 0.001) (see Table 1). In particular, the right ventricle reduced in volume, with a significant reduction in the population with moderate-to-severe dilatation (from 89% to 44%) with a respective increase to the proportion of normal volumes (from 11% to 56%). The acute right ventricular volume reduction affected the function that was mildly reduced in 68% of the population. The tricuspid valve regurgitation was moderate in 11% of the population and decreased to 3% after repair. The improvement in echocardiographic parameters was significant and similar to all three approaches used (Table 1), suggesting equal efficacy in the surgical repair of the MRAT compared to SMS. All patients were discharged in good clinical condition, without significant intracardiac defects or extracardiac impairment; no significant surgical wound infections were observed.

## 4. Discussion

In recent years, interest in improving cosmetics and reducing the psychological implications of cardiac surgery has assumed increased importance, with particular attention paid to young patients [11,15]. As a result, a variety of minimally invasive surgical techniques have been developed for cardiac surgery in the pediatric population, including right mini-thoracotomy [12]. In the current era, the trans-catheter approach has evolved and gained more importance in ASD closure as a great alternative to surgery because of the short post-procedural recovery and the cosmetic outcomes [16,17]. Many recent studies have reported no mortality and a very low rate of major complications (<1% of patients) [18,19]. Recently, the device closure of ASD has become the first choice for the correction of ASDs, but the lack of an anatomical device landing border or the presence of other associated lesions may not allow percutaneous treatment. Minimally invasive techniques provide good repair results with an early return to normal daily activity and a physically and psychologically high-quality life after surgery [3,16]. Many studies showed that the minimally invasive approach is safe and represents a good option for correcting simple congenital heart defects, providing better cosmetic results and non-inferior clinical outcomes compared to the conventional approach to median sternotomy [7,8,15]. Although minimally invasive functional advantages are widely recognized, some of these approaches have limited indications in the pediatric field due to suboptimal cosmetic results (i.e., mini-sternotomy, right anterolateral thoracotomy). For example, in the long-term follow-up, the right anterolateral thoracotomy was associated with reduced volume in the right breast compared to median sternotomy. Therefore, right anterolateral thoracotomy has been limited only to adult female patients whose breasts have already developed [20,21].

The MRAT allows the concealment of the incision beneath the right arm and avoid the spares of the sternum. The lateralization of the incision offers other advantages because it does not involve the breast tissue, abolishes mammary gland lesions in prepubescent women, and prevents the impairment of breast development and chest wall deformities [22]. An efficient adoption of the MRAT requires a standardized program that reduces the learning curve, guarantees low morbidity, and maintains patient safety. For this reason, in 2017, we decided to start the MRAT program with the supervision and tutoring of an expert in the field to provide insights to achieve immediate good results and excellent safety. With this program, we were able to reduce learning curves at about 15 consecutive procedures per surgeon to reach the cardiopulmonary bypass and surgical operative times similar to median sternotomy. In terms of efficacy, despite being a newly started program, there were no major post-operative complications or major cases of morbidity compared to sternotomy or minimal sternotomy.

We conduct yearly follow-ups for patients with simple CHD, such as the ones treated in this study. Considering this, to assess the full advantage of the esthetical impacts on patients, we need to wait adolescents age, especially young females who still have not completed their breast development. So far, we have collected reports of parents’ satisfaction that are all positive, although patient satisfaction can not be fully comprehended due to different factors, such as the patients’ young ages and incomplete development in the majority of the population. In the future, we aim to conduct a questionnaire directed to patients to investigate satisfaction with the approach and the psychological impact of scarring below the arm or in the middle of the sternum. Finally, surgical repair of ASD, pAVSD, or PAPVD through MRAT allows safe repair of the defect with similar echocardiographic recovery of the heart compared to SMS. In particular, MRAT, similarly to SMS, guarantees post-surgical reduction of the right volume overload, reduces right ventricle dilatation, and guarantees similar atrioventricular valve improvement. Therefore, the MRAT is not only comparable to SMS in terms of surgical safety but is also comparable in terms of patient clinical and instrumental post-operative recovery. It is fundamental to note that, although such data were not collected directly, parents and patients expressed great satisfaction regarding the cosmetic results due to the fact that skin incisions were not immediately visible.

## 5. Conclusions

The surgical repair of simple congenital heart anomalies, such as atrial septal defects or partial anomalous pulmonary venous returns, can be achieved through minimal right axillary thoracotomies, which guarantee safety and efficacy without increasing surgical risk or the duration of surgery. Minimal right axillary thoracotomy preserves the sternum from surgical fractures and allows the concealment of surgical scars beneath the arm, preserving the patient’s aesthetic qualities.

## Figures and Tables

**Table 1:** Pre-operative and discharge echocardiographic data. Pre-operative and discharge data comparisons for each single approach (intended as a shift from the pre-operative to the post-operative value) were assessed with symmetry test (Bowker’s test and the Stuart–Maxwell test). A significant *p*-value indicates that there has been a significant shift between categories of the variable.

	MRAT (n = 78 pts)					Sternotomy (n = 138 pts)				
Variable	Pre-Operative n (%)		Post-Operative n (%)		*p*-Value	Pre-Operative n (%)		Post-Operative n (%)		*p*-Value
**Left ventricle EF**					ns					0.1573
Normal	78	100%	78	100%		138	100%	138	100%	
**Right ventricle EF**					0.0009					0.0009
Normal	78	100%	33	42%		138	100%	61	44%	
**Right ventricle dilatation**					0.0009					0.0009
No	1	1%	26	33%		9	7%	36	26%	
Mild	6	8%	17	22%		8	6%	41	30%	
Moderate	61	78%	34	44%		97	70%	60	43%	
Severe	10	13%	1	1%		24	17%	0	0%	
**Pulmonary artery pressure increment**					0.0558					0.0001
Normal	67	86%	75	96%		101	73%	126	91%	
Mild	7	9%	3	4%		24	17%	10	7%	
>Moderate	4	5%	0	0%		13	9%	1	1%	
**Tricuspid valve regurgitation degree**					0.2719					0.0009
No	15	19%	23	29%		13	9%	36	26%	
Mild	57	73%	51	65%		107	78%	100	72%	
Moderate	5	6%	4	5%		16	12%	2	1%	
Severe	0	0%	0	0%		2	1%	0	0%	
**Mitral valve regurgitation degree**					0.2035					0.1395
Normal	69	88%	66	85%		119	86%	124	90%	
Mild	4	5%	11	14%		12	9%	13	9%	
Moderate	4	5%	1	1%		7	5%	1	1%	

**Table 2:** Type of cannulation according to different approaches.

Type of Cannulation					
	MRAT (n)	SMS (n)	SMS Mini (n)	Total n (%)	
Central	65	116	21	202	(93.5%)
Peripheral	7	0	1	8	(3.7%)
Mixed	6	0	0	6	(2.8%)
Total	78	116	22	216	(100%)

**Table 3:** Population demographic, operative and post-operative. Data expressed as median and (IQR). *p*-values calculated with Kruskal–Wallis test and, if significant, * Dunn’s test with Bonferroni adjustment for differences among subgroups with significant difference only between MRAT vs. SMS.

Variable	MRAT (n = 78)	SMS (n = 116)	SMS Mini (n = 22)	Total	*p*-Value	MRAT vs. SMS *
Age (years)	5.0 (4)	9.5 (18.5)	6.5 (6)	6.1 (10)	0.0001	0.00009
Weight (kg)	18.3 (13)	30 (40)	23 (22)	22 (35)	0.0002	0.0001
Height (cm)	110 (24)	140 (54)	122 (40)	122 (52)	0.0001	0.00009
CPB time (min)	55 (23)	49 (32)	45.5 (20)	52 (28)	0.06	n.s.
Aortic cross-clamp (min)	27 (14)	29 (25)	24 (13)	27 (19)	0.6741	n.s.
Surgical Time (min)	190 (61)	170 (60)	170 (34)	178 (64)	0.0133	0.0062
ICU stay (days)	1 (0)	1 (0)	1 (0)	1 (0)	0.7265	n.s.
Hospital stay (days)	7 (1)	7 (4)	6 (1)	7 (3)	0.0036	0.0057
CPB lowest HTC (%)	28 (5)	29 (4)	29 (4)	28 (5)	0.0062	0.0027
CPB lowest temp (C)	34.6 (1)	34.2 (1.2)	34.0 (1.6)	34.3 (1)	0.8304	n.s.
CPB highest Lactate	1.1 (0.4)	1.0 (0.5)	1.1 (0.5)	1.1 (0.4)	0.995	n.s.

## Data Availability

The raw data supporting the conclusions of this article will be made available in an online platform (Zenodo) and made available upon request.
